# Coping Strategies in Liver Transplant Recipients and Caregivers According to Patient Posttraumatic Growth

**DOI:** 10.3389/fpsyg.2017.00018

**Published:** 2017-01-20

**Authors:** M. Ángeles Pérez-San-Gregorio, Agustín Martín-Rodríguez, Mercedes Borda-Mas, M. Luisa Avargues-Navarro, José Pérez-Bernal, M. Ángel Gómez-Bravo

**Affiliations:** ^1^Department of Personality, Assessment, and Psychological Treatment, Faculty of Psychology, University of SevilleSeville, Spain; ^2^Critical Care and Urgencies, University Hospital Virgen del Rocío of SevilleSeville, Spain; ^3^Hepatic-Biliary-Pancreatic Surgery and Liver Transplant Unit, University Hospital Virgen del Rocío of SevilleSeville, Spain

**Keywords:** liver transplantation, posttraumatic growth, coping strategies, patients, caregivers

## Abstract

The purpose of this study was to analyze the differences in coping strategies employed by liver transplant recipients and their family members according to patient posttraumatic growth. Two matched groups of 214 liver transplant recipients and 214 family members were selected. The Posttraumatic Growth Inventory and Brief COPE were used. The most relevant results were: (1) Interactive effects in active coping, support (instrumental and emotional) and acceptance strategies, which were all used more by patients with higher growth levels, while their family members showed no differences in use of these strategies by patient growth level. Furthermore, while a low level of patient growth did not mark differences between them and their caregivers, a high level did, patients employing more active coping and support (instrumental and emotional), (2) In both groups a high level of patient growth was associated with more use of positive reframing and denial than a low one, and (3) Self-blame was employed by patients more than by their caregivers. It was concluded that a high level of posttraumatic growth in liver transplant recipients is associated with more use of healthy coping strategies, basically active coping, instrumental support, and emotional support.

## Introduction

Liver transplantation is a therapeutic option which increases the patient’s quality of life, although not up to normative data (Fernández-Jiménez et al., 2012; Pérez-San-Gregorio et al., 2013). Such surgery forms part of a very stressful process which must be faced by the patients and their families. The period before the transplant is marked by uncertainty, specifically, the unknown about whether the patient will meet the requirements for getting on the waiting list or how long he will have to be on it until a compatible donor becomes available (Meltzer and Rodrigue, 2001; Martín-Rodríguez et al., 2014). The post-transplant stage is characterized by patient survival depending on a lifelong immunosuppressant treatment, with constant medical checkups and tests, in addition to a strong fear of losing the implant and other medical complications such as recurrence (Pérez-San-Gregorio et al., 2012; Reed-Knight et al., 2013). Thus immunosuppressant treatment has secondary effects with negative repercussions on the patient’s quality of life (Grinyó et al., 2012). This can be coped with in different ways, from strategies facilitating adaptation to a new medical condition (for example, acceptance or positive reframing) to other strategies which would impede it. All this time, family members take on the role of caregivers, so they provide the patient with emotional support, take them to doctors’ appointments, look out for their dietary requirements, and do housework (Cohen et al., 2007). These caregivers may suffer from the frustration of their professional plans, employment complications, economic difficulties and even conflictive relations between the patients and their caregivers (Miyazaki et al., 2010).

Living under these conditions is associated with a diversity of psychological complications for both patients and their caregivers which usually appear in the post-transplant stage: anxiety, depression, feelings of blame, fantasies about the donor, excessive gratitude toward the donor’s family, etc. (Pérez-San-Gregorio et al., 2005; Grover and Sarkar, 2012; Látos et al., 2012; Errichiello et al., 2014). Nevertheless, these complications may coexist with posttraumatic growth, which refers to a series of positive changes experienced by the person as a result of the struggle ensuing experience of a traumatic event (Tedeschi and Calhoun, 1996). In the scope of transplantation, some studies on cancer patients who have received a hematopoietic stem cell transplant have found high levels of posttraumatic growth, where these patients appreciated more value in their own lives, changed the priorities of what is important in their lives, appreciated each day more, learned how wonderful other people are and realized that they could count on them in times of trouble (Widows et al., 2005; Tallman et al., 2010).

Therefore, because of its repercussions on clinical practice, it is of great interest to find out whether the coping strategies of liver transplant recipients are different from those of their caregivers, and the influence patient posttraumatic growth could have on these differences. In spite of its relevance, there are no such studies in the field of liver transplantation, although in other clinical samples an association has been found between posttraumatic growth and certain coping strategies. For example, in patients with spinal cord injuries, it has been associated with mental disengagement and active coping (Pollard and Kennedy, 2007), in patients with rheumatoid arthritis or those who have had a myocardial infarction, it is associated with problem-focused coping (Dirik and Karanci, 2008; Senol-Durak and Ayvasik, 2010), in bone marrow transplant recipients, it is associated with avoidance, positive reinterpretation and problem-solving (Widows et al., 2005), and in cancer patients it is associated with active coping, positive reevaluation and religion (Schmidt et al., 2012; Shand et al., 2015). Some of these studies demonstrate that both avoidance and problem-focused strategies are important to posttraumatic growth (Ogińska-Bulik and Kobylarczyk, 2015). However, other studies in cancer patients have concluded that avoidance strategies (denial, behavioral disengagement, and self-blame) are unrelated to positive changes (Park et al., 2008) and even that strategies such as self-distraction are related to negative changes (Schroevers et al., 2011).

The transplant definitely involves confronting exceptional life events. This could lead to various levels of posttraumatic growth in patients associated with the use of different coping strategies. As there is no clear causal relationship between coping strategies and posttraumatic growth, and in other clinical samples the results are contradictory, in this study, we analyzed the difference in coping strategies used by liver transplant recipients and their caregivers as a function of patient posttraumatic growth (low, medium, and high).

## Materials and Methods

### Participants

Two matched groups were selected for a 2 × 3 mixed factorial design: 214 liver transplant recipients with different levels of posttraumatic growth (low, medium, and high) and 214 family members (each patient’s main caregiver). The design had two independent variables, one intra referring to group with two values (patient and caregiver), and another inter referring to posttraumatic growth of the patients with three values (low, medium, and high), depending on the patient’s total score on the Posttraumatic Growth Inventory.

The transplant patient group (G1) was made up of 165 men and 49 women with a mean age of 60.41 years (*SD* = 9.36 years). The liver in all cases came from a donor who had died from one of the following causes: stroke (60.7%), traumatic brain injury (27%) and others (12.3%). **Table 1** shows the sociodemographic and clinical characteristics of the three of liver transplant subgroups.

**Table 1:** Comparison of the sociodemographic and clinical variables of the three subgroups of liver transplant recipients (G1) with different levels of posttraumatic growth (low, medium, and high).

	Posttraumatic growth levels			Intergroup comparisons		Effect sizes
			
	Low	Medium	High			
	*n* = 70	*n* = 71	*n* = 73			
	a	b	c			

	***M* (*SD*)**	***M*** **(*SD*)**	***M* (*SD*)**	***F*(2.211)**	***p***	**Cohen’s *d***
Age	61.54 (8.85)	59.89 (9.59)	59.82 (9.62)	0.77	0.466	
					a–b = 0.547	0.18 N
					a–c = 0.516	0.19 N
					b–c = 0.999	0.01 N
Time since transplant (in months)	79.76 (63.44)	95.63 (67.96)	88.41 (71.57)	0.97	0.381	
					a–b = 0.348	-0.24 S
					a–c = 0.726	-0.13 N
					b–c = 0.799	0.10 N

	**%**	**%**	**%**	**χ^2^**	***p***	**Cohen’s *w***

Gender				1.07	0.586	0.07 N
- Male	30.9	33.9	35.2			
- Female	38.8	30.6	30.6			
Marital status				0.63	0.731	0.05 N
- Partner	32.6	34.3	33.1			
- No partner	33.3	28.2	38.5			
Education				7.32	0.120	0.18 S
- Low	28.7	32.4	39.0			
- Medium	35.6	31.1	33.3			
- High	45.5	39.4	15.2			
Employment				1.94	0.379	0.09 N
- Working	43.8	37.5	18.8			
- Not working	31.8	32.8	35.4			
Etiology of the liver disease				13.28	0.103	0.25 S
- Alcoholic	29.4	30.9	39.7			
- Hepatocellular carcinoma	33.3	25.0	41.7			
- Hepatitis C virus	32.4	43.2	24.3			
- Hepatitis B virus	8.3	50.0	41.7			
- Others	45.9	35.1	18.9			


*N, null effect size; S, small effect size.*

The caregiver group (G2) was made up of 47 men and 167 women with a mean age of 53.19 years (*SD* = 12.62 years). The relationship to the patients was: partner (71%), child (19.6%), sibling (4.2%), mother/father (3.7%), or other (1.5%).

### Instruments

The Posttraumatic Growth Inventory (Tedeschi and Calhoun, 1996) consists of 21 items answered on a Likert-type scale from 0 (“no change”) to 5 (“very great degree of change”) evaluating perception of personal benefits in survivors of traumatic events. It provides information referring to the total score on the scale and five dimensions: relating to others, new possibilities, personal strength, spiritual change and appreciation of life. We used the Spanish version provided by Weiss and Berger (2006). This inventory was administered to patients, with a Cronbach’s alpha in this study of 0.94 for the complete scale and 0.73–0.88 on the various subscales.

The Brief COPE (Carver, 1997) is made up of 28 items answered on a Likert-type scale from 0 (“no, not at all”/“I haven’t been doing this at all”) to 3 (“a lot”/“I’ve been doing this a lot”). It evaluates 14 coping strategies: active coping, planning, instrumental support, emotional support, self-distraction, venting, disengagement, positive reframing, denial, acceptance, religion, substance use, humor, and self-blame. We used the Spanish version provided by Morán et al. (2010). This scale was administered to the patients and their caregivers, with a Cronbach’s alpha in this study of 0.50 to 0.85 on the various subscales, except for the disengagement subscale which not surpass 0.50.

### Procedure

After this study was approved by the Ethics Committee of the Virgen del Rocío University Hospital of Seville, we selected two matched groups of liver transplant recipients and their family members.

To select the transplant group, we started out from a clinical population of all those patients who had received a liver transplant from a deceased donor in Seville from 1990 to 2014. During this period, 1053 liver transplantations were performed in adults, of which only 569 patients remained alive at the start of this study. All of them and their caregivers were informed through the Association of Liver Transplant Recipients and the Hepatic-Biliary-Pancreatic Surgery and Liver Transplant Unit of the possibility of participating in this psychological study.

The criteria for inclusion in both groups were that they must be of adult age, sign the informed consent, have no problem understanding the evaluation instruments employed, and not have any severe pathology or disability at the time of evaluation which would impede comprehension of the items. Other considerations specific to patients were whether a family member was caring for the patient (accompanied them to checkups and medical tests, supervised their immunosuppressant medication, etc.) and whether they had only had one transplant.

Based on these criteria, 331 patients were excluded: 282 because they did not wish to participate, two could not be located, 15 did not understand the instruments, eight had other disorders (stroke, hepatic encephalopathy, etc.) and 24 were retransplant patients, leaving 238 patients finally evaluated. Of the main caregivers of each of these patients, 12 did not wish to participate, two did not understand the instruments, and 10 patients did not have a main caregiver. Therefore, 214 patient-caregiver pairs were finally evaluated.

### Statistical Analysis

Data were analyzed using the SPSS 22 statistics program. Pearson’s Chi-Square was used to compare the qualitative variables in the three patient subgroups, and to compare the quantitative variables, a one-way ANOVA was applied with the Tukey HDS test for *post hoc* comparison. A 2 × 3 mixed factorial ANCOVA was also done to evaluate the influence on coping strategies exerted by group (liver transplant recipients and caregivers) and patient posttraumatic growth level (low, medium, or high). Time since transplantation (in months) was included as a covariate in this analysis. The Cohen’s *w* (for qualitative variables) and Cohen’s *d* (for quantitative variables) were calculated as an index of effect size.

## Results

Three groups were formed based on the total score by liver transplant recipients on the Posttraumatic Growth Inventory: (a) 70 patients with low posttraumatic growth (32.7% of the sample, 0–57 points), (b) 71 patients with medium posttraumatic growth (33.2% of the sample, 58–77 points), and (c) 73 patients with high posttraumatic growth (34.1% of the sample, 78–105 points). Two criteria were used for the selection of these three groups: (1) the total scores found for each of the patients on the Posttraumatic Growth Inventory, which varied from 0 to 105, and (2) after putting patient scores in order from lowest to highest, three subgroups were formed corresponding to about one third of the sample based on the percentiles found. As shown in **Table 1**, among the three subgroups there were no differences in sociodemographic or clinical variables (*d*s from 0.01 to 0.25, null or small effect sizes).

**FIGURE 1:**
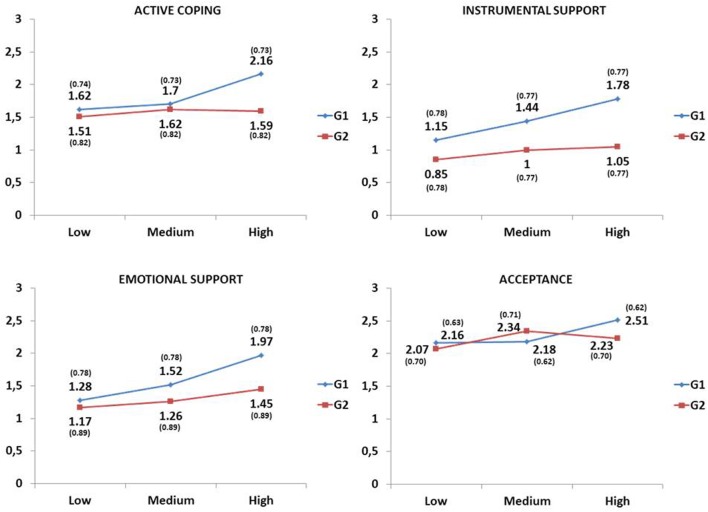
**Interactive effects of the group and patient posttraumatic growth level (low, medium, and high) factors.** Means (standard deviations) adjusted for time since transplant covariate, G1 = Liver transplant recipients, G2 = Caregivers.

Interactive effects between the two factors in the study were found in the following coping strategies: active coping [*F*(2,210) = 5.30, *p* = 0.006], instrumental support [*F*(2,210) = 4.21, *p* = 0.016], emotional support [*F*(2,210) = 3.29, *p* = 0.039], and acceptance [*F*(2,210) = 4.29, *p* = 0.015]. Specifically, as shown in **Figure 1** and **Tables 2** and **3**, the simple effects demonstrated that these four coping strategies were more used by the patients with the highest levels of posttraumatic growth (*d*s from -0.55 to -0.89, medium or large effect sizes), while there was no variation in the use of these strategies for caregivers as a function of posttraumatic growth of patients (*d*s from 0.04 to -0.38, null or small effect sizes) (**Table 2**). Furthermore, while low posttraumatic growth in patients did not mark important differences between them and their caregivers (*d*s from 0.13 to 0.38, null or small effect sizes), a high level did show very relevant differences in which patients employed active coping (*p* < 0.001, *d* = 0.74, medium effect size), instrumental support (*p* < 0.001, *d* = 0.95, large effect size) and emotional support (*p* < 0.001, *d* = 0.62, medium effect size) strategies more than their caregivers. Medium posttraumatic growth also showed a difference in instrumental support (*p* < 0.001, *d* = 0.56, medium effect size), as this strategy was more used by patients than by their caregivers (**Table 3**).

**Table 2:** Simples effects: comparisons of patient posttraumatic growth levels in liver transplant recipient (G1) and caregiver (G2) groups.

	G1 *n* = 214		G2 *n* = 214	
		
Posttraumatic growth levels (G1)	*p*	Cohen’s *d*	*p*	Cohen’s *d*
*Active coping*				
Low-medium	1.000	-0.10 N	1.000	-0.14 N
Low-high	<0.001	-0.73 M	1.000	-0.09 N
Medium-high	0.001	-0.63 M	1.000	0.04 N
*Instrumental support*				
Low-medium	0.075	-0.38 S	0.727	-0.20 S
Low-high	<0.001	-0.81 L	0.414	-0.25 S
Medium-high	0.030	-0.43 S	1.000	-0.05 N
*Emotional support*				
Low-medium	0.198	-0.31 S	1.000	-0.10 N
Low-high	<0.001	-0.89 L	0.195	-0.31 S
Medium-high	0.002	-0.57 M	0.625	-0.21 S
*Acceptance*				
Low-medium	1.000	-0.04 N	0.071	-0.38 S
Low-high	0.003	-0.55 M	0.532	-0.23 S
Medium-high	0.007	-0.52 M	1.000	0.16 N


*N, null effect size; S, small effect size; M, medium effect size; L, large effect size; G1, liver transplant recipients; G2, caregivers.*

**Table 3:** Simples effects: comparisons of liver transplant recipients (G1) and their caregivers (G2) at each of the patient posttraumatic growth levels.

Posttraumatic growth levels	Active coping		Instrumental support		Emotional support		Acceptance	
				
	*p*	Cohen’s *d*	*p*	Cohen’s *d*	*p*	Cohen’s *d*	*p*	Cohen’s *d*
Low	0.335	0.15 N	0.009	0.38 S	0.363	0.13 N	0.401	0.13 N
Medium	0.523	0.10 N	<0.001	0.56 M	0.026	0.31 S	0.134	-0.24 S
High	<0.001	0.74 M	<0.001	0.95 L	<0.001	0.62 M	0.009	0.41 S


*N, null effect size; S, small effect size; M, medium effect size; L, large effect size*

Concerning the main effects, the posttraumatic growth factor was significant in positive reframing (*p* = 0.006) and denial (*p* = 0.008) coping strategies, where a high level of posttraumatic growth showed an important association with greater use of positive reframing (*p* = 0.004, *d* = -0.54, medium effect size) and denial (*p* = 0.006, *d* = -0.53, medium effect size) than a low one. The group factor also marked an import difference in the self-blame strategy (*p* < 0.001, *d* = 0.55, medium effect size), which was used much more by patients than by their caregivers (**Table 4**).

**Table 4:** Coping strategies: differences between liver transplant recipients (G1) and their caregivers (G2) by patient posttraumatic growth levels.

	Posttraumatic growth levels *M*^1^ (*SD*)			Comparisons *p* (Cohen’s *d*)			Groups *M*^1^ (*SD*)		Main effects		Interactive effects
					
	Low	Medium	High	a–b	a–c	b–c	G1	G2	Posttraumatic	Groups *F*(1,210) (*p*)	*F*(2,210) (*p*)
	*n* = 70	*n* = 71	*n* = 73				*n* = 214	*n* = 214	growth levels	[Cohen’s *d*]	
	a	b	c						*F*(2,210) *(p)*		
Planning	1.12	1.38	1.26	1.000	0.536	0.716	1.23	1.11	1.09	4.17	1.42
	(0.63)	(0.63)	(0.62)	(-0.03 N)	(-0.22 S)	(-0.20 S)	(0.85)	(0.73)	(0.338)	(0.036) [0.16 N]	(0.245)
Self-distraction	0.85	1.06	1.13	0.234	0.062	1.000	1.07	0.95	2.95	0.28	1.27
	(0.72)	(0.72)	(0.72)	(-0.30 S)	(-0.39 S)	(-0.09 N)	(0.89)	(0.86)	(0.054)	(0.598) [0.14 N]	(0.282)
Venting	0.73	0.76	0.76	1.000	1.000	1.000	0.76	0.74	0.05	0.01	1.93
	(0.61)	(0.61)	(0.61)	(-0.05 N)	(-0.05 N)	(0.00 N)	(0.75)	(0.75)	(0.952)	(0.915) [0.02 N]	(0.147)
Disengagement	0.22	0.26	0.23	1.000	1.000	1.000	0.34	0.13	0.24	5.69	0.09
	(0.35)	(0.34)	(0.35)	(-0.11 N)	(-0.04 N)	(0.07 N)	(0.55)	(0.35)	(0.784)	(0.018) [0.45 S]	(0.913)
Positive reframing	1.28	1.48	1.62	0.174	0.004	0.560	1.46	1.46	5.31	0.80	2.71
	(0.63)	(0.63)	(0.62)	(-0.32 S)	(-0.54 M)	(-0.22 S)	(0.79)	(0.77)	(0.006)	(0.373) [0.00 N]	(0.069)
Denial	0.28	0.40	0.53	0.369	0.006	0.348	0.46	0.35	4.92	0.16	2.05
	(0.48)	(0.48)	(0.48)	(-0.26 S)	(-0.53 M)	(-0.26 S)	(0.63)	(0.60)	(0.008)	(0.686) [0.17 N]	(0.131)
Religion	1.00	1.01	1.34	1.000	0.023	0.029	1.07	1.16	4.73	3.60	1.00
	(0.76)	(0.76)	(0.76)	(-0.01 N)	(-0.45 S)	(-0.44 S)	(0.98)	(0.95)	(0.010)	(0.059) [-0.09 N]	(0.371)
Substance use	0.05	0.05	0.05	1.000	1.000	1.000	0.05	0.05	0.00	0.76	0.39
	(0.21)	(0.21)	(0.21)	(0.01 N)	(0.00 N)	(-0.01 N)	(0.29)	(0.23)	(0.996)	(0.384) [0.03 N]	(0.679)
Humor	0.49	0.48	0.49	1.000	1.000	1.000	0.57	0.41	0.02	1.19	1.03
	(0.58)	(0.59)	(0.59)	(0.02 N)	(-0.01 N)	(-0.03 N)	(0.77)	(0.69)	(0.983)	(0.277) [0.22 S]	(0.360)
Self-blame	0.51	0.64	0.56	0.490	1.000	1.000	0.76	0.38	0.99	14.21	0.68
	(0.54)	(0.54)	(0.54)	(-0.24 S)	(-0.10 N)	(0.14 N)	(0.80)	(0.57)	(0.374)	(<0.001) [0.55 M]	(0.507)


*^1^Means adjusted for the time since transplant covariate; N, null effect size; S, small effect size; M, medium effect size; G1, liver transplant recipients; G2, caregivers.*

## Discussion

The most relevant finding of this study was the interactive effects of the active coping, instrumental support, emotional support and acceptance variables. All of them were used to a greater extent by liver transplant recipients who had more posttraumatic growth (medium or large effect sizes). However, the use of those strategies by family members did not vary with patient posttraumatic growth level (null or small effect sizes). This means that patients with more posttraumatic growth face their problems by employing adaptive strategies, that is, they take action or carry out specific activities to solve their problems (active coping), seek advice and information about what they should do (instrumental support), consolation and understanding from others (emotional support), and also recognize the problems they are going through (acceptance). This result on acceptance supports Zoellner and Maercker’s (2006) theory on the importance of accepting traumatic situations, usually life events which are uncontrollable or unchangeable, in the process of posttraumatic growth. The other three strategies (active coping, instrumental support, and emotional support), while low-level posttraumatic growth did not mark differences between patients and their caregivers (null or small effect sizes), the opposite was true of high-level posttraumatic growth, which did mark relevant differences (medium or large effect sizes) and were more used by the patients than by caregivers. This difference with the same trend was also observed in the instrumental support strategy when patients had a medium posttraumatic growth level. A possible explanation for these results, as demonstrated in breast cancer patients, would be that these strategies foster positive changes in liver transplant recipients. This means that the active effort to try and change difficult circumstances for the better, facing the problem, and seeking support, would increase the possibility that the patients have fewer negative feelings when they can express them. This would therefore allow them to concentrate on the benefits, which along with the increase in close relationships, would contribute to recognition of one’s own personal strength (Manne et al., 2004). In this same line, the fact that patients with more posttraumatic growth employ active coping to face their problems has also been confirmed in other studies done with patients suffering from rheumatoid arthritis (Dirik and Karanci, 2008), have had spinal cord injury (Pollard and Kennedy, 2007), heart attack (Senol-Durak and Ayvasik, 2010) or breast cancer (Schmidt et al., 2012). Seeking support (instrumental and emotional) as a coping strategy provides an opportunity for patients to tell their problems to someone and express their emotions and negative thoughts. This facilitates event processing, trying to understand it, and finding a positive meaning in it (Cordova et al., 2001; Manne et al., 2004). Thus, different types of support (emotional, cognitive, and instrumental) are associated with posttraumatic growth (Hasson-Ohayon et al., 2016).

Another very interesting result was that regardless of the role (patient or caregiver), a high level of patient posttraumatic growth was associated with more use of positive reframing and denial than a low level. Along this same line, in bone marrow transplant (Widows et al., 2005) and cancer patients (Schmidt et al., 2012; Shand et al., 2015), posttraumatic growth was also associated with positive reframing. It could be said that the effort patients and caregivers make to interpret the threat positively would facilitate the search for real positive changes, or at least promote the perception that such changes have occurred (Thornton and Perez, 2006). With regard to denial, similar results were also found in bone marrow transplant recipients, which could be explained because under some circumstances, taking on an active attitude toward problems would facilitate posttraumatic growth, while in others, the use of denial would alleviate the anxiety temporarily, leading in turn to the perception of growth (Widows et al., 2005; Dirik and Karanci, 2008). In fact, as argued by Ogińska-Bulik and Kobylarczyk (2015), both avoidance and problem-focused strategies are relevant in posttraumatic growth. Similarly, although without controlling for the influence of posttraumatic growth, Stilley et al. (2010) found that 17% of liver transplant recipients used both approach and avoidance strategies to face the various problems related to transplantation.

On the other hand, being a patient or a caregiver made an important difference (medium effect size) in the use of the self-blame strategy regardless of posttraumatic growth of the patient. It is the patients, rather than their family members, who tend to criticize and blame themselves for what happened. This result could be explained by patients considering themselves responsible for the distress their family members feel due to their illness, and feeling like a burden on the whole family (Domínguez-Cabello et al., 2012a). It should be stressed that the main illnesses triggering liver transplant are alcohol addiction and illegal use of drugs, reason for which, for example, the caregiver of a patient who is a candidate for a liver transplant compared to those of a lung transplant, experience a heavier burden (Meltzer and Rodrigue, 2001), in addition to suffering in each of the stages associated with transplantation, especially in those before it (pre-transplant study and waiting list) (Domínguez-Cabello et al., 2012b). In other etiologies leading to liver transplant, such as hepatitis C virus, patients have been described as socially isolating themselves, whether they received a transplant or not, mainly for fear of transmitting the virus to family and friends, which generates feelings of guilt and shame (Dudley et al., 2007; Pérez-San-Gregorio et al., 2012). To all of the above it would have to be added that transplant patients sometimes feel guilty for the death of their donor to the point of having difficulties in organ acceptance (Zimbrean, 2015). Regardless of the patient’s posttraumatic growth, the main difference from their caregivers is that the latter are less self-critical. This result is very significant, as it would reduce the caregiver’s burden by presenting less guilt for all the circumstances associated with the transplant.

Summarizing, the main contribution of this study is that a higher level of posttraumatic growth in liver transplant recipients is associated with their using more adaptive coping strategies compared to their family members to face their problems. Therefore, and as suggested by Dirik and Karanci (2008), it would be very advisable to strengthen healthy coping strategies to be able to reinforce posttraumatic growth. It would also be beneficial to increase posttraumatic growth by means of intervention based, among others, on self-help groups or cognitive-behavioral therapy, as demonstrated in cancer patients who undergo hematopoietic stem cell transplantation (Jeon et al., 2015).

Finally, some limitations of this study which should be considered in future research would be the following: (1) The results of the comparison of coping strategies between patients and their caregivers could vary depending on the levels of posttraumatic growth of the family member, (2) Posttraumatic growth and some coping strategies (for example, positive reframing) might overlap, and (3) finally, to solve the specific causal relationship between posttraumatic growth and coping strategies, longitudinal studies would be advisable, as both aspects could vary over time.

## Author Contributions

MÁP-S-G, AM-R, MB-M, MLA-N, JP-B, and MÁG-B: Conception and design of the work; data analysis and interpretation; revising the article critically for important intellectual content; giving final approval of the version to be submitted. MÁP-S-G and AM-R: Bibliography research about the topic; data collection; drafting the article.

## Conflict of Interest Statement

The authors declare that the research was conducted in the absence of any commercial or financial relationships that could be construed as a potential conflict of interest.
